# The frontline of immune response in peripheral blood

**DOI:** 10.1371/journal.pone.0182294

**Published:** 2017-08-03

**Authors:** Fuhai Song, Ying Qian, Xing Peng, Xiuhui Li, Peiqi Xing, Dongqing Ye, Hongxing Lei

**Affiliations:** 1 CAS Key Laboratory of Genome Sciences and Information, Beijing Institute of Genomics, Chinese Academy of Sciences, Beijing, China; 2 Cunji Medical School, University of Chinese Academy of Sciences, Beijing, China; 3 Center of Alzheimer’s Disease, Beijing Institute for Brain Disorders, Beijing, China; Universitatsklinikum Hamburg-Eppendorf, GERMANY

## Abstract

Peripheral blood is an attractive source for the discovery of disease biomarkers. Gene expression profiling of whole blood or its components has been widely conducted for various diseases. However, due to population heterogeneity and the dynamic nature of gene expression, certain biomarkers discovered from blood transcriptome studies could not be replicated in independent studies. In the meantime, it’s also important to know whether a reliable biomarker is shared by several diseases or specific to certain health conditions. We hypothesized that common mechanism of immune response in blood may be shared by different diseases. Under this hypothesis, we surveyed publicly available transcriptome data on infectious and autoimmune diseases derived from peripheral blood. We examined to which extent common gene dys-regulation existed in different diseases. We also investigated whether the commonly dys-regulated genes could serve as reliable biomarkers. First, we found that a limited number of genes are frequently dys-regulated in infectious and autoimmune diseases, from which we selected 10 genes co-dysregulated in viral infections and another set of 10 genes co-dysregulated in bacterial infections. In addition to its ability to distinguish viral infections from bacterial infections, these 20 genes could assist in disease classification and monitoring of treatment effect for several infectious and autoimmune diseases. In some cases, a single gene is sufficient to serve this purpose. It was interesting that dys-regulation of these 20 genes were also observed in other types of diseases including cancer and stroke where certain genes could also serve as biomarkers for diagnosis or prognosis. Furthermore, we demonstrated that this set of 20 genes could also be used in continuous monitoring of personal health. The rich information from these commonly dys-regulated genes may find its wide application in clinical practice and personal healthcare. More validation studies and in-depth investigations are warranted in the future.

## Introduction

Peripheral blood as a minimally invasive source has been widely used in biomarker discovery for many diseases. Due to the richness in gene expression information, blood transcriptome has become a primary source for blood-derived biomarkers. In a pioneering work, unsupervised clustering of transcriptome data was able to separate acute myeloid leukemia (AML) from acute lymphoblastic leukemia (ALL) with high accuracy[1]. Blood transcriptome studies have also been applied to several solid tumors, including the early detection of breast cancer[2], stage classification of colorectal cancer[3], prognosis of castration-resistant prostate cancer[4], and gene signature for treatment response in nasopharyngeal carcinoma[5].

Due to the direct link between blood and the immune system, many studies on the blood transcriptome have been focused on infectious or autoimmune diseases. A major concern in biomarker discovery is the reproducibility in independent studies. To obtain reliable biomarkers, different approaches have been reported in the literature. For example, clustering of differentially expressed genes followed by machine learning have been applied to the discrimination of three subclasses of septic shock [6]. However, machine learning approaches are prone to over-fitting if not carefully utilized. To achieve better reproducibility in independent studies, a two-stage design may be applied on multiple datasets, where the model derived from the discovery datasets are validated in additional independent datasets. In one such study, a panel of 11 genes were found to be able to discriminate sterile inflammation from infectious inflammation[7]. Another way to enhance the prediction power is to use gene modules rather than individual genes, which has demonstrated good performance on systemic lupus erythematosus (SLE) and other diseases[8].

To enhance the reproducibility of biomarker, we could also select genes consistently dys-regulated in multiple independent studies on different diseases, which further reduce the likelihood that the observed gene dys-regulation is due to population heterogeneity or transient fluctuation. We hypothesized that similar immune response may be induced in peripheral blood in different infectious and autoimmune diseases, or even in other diseases such as cancer and neurological disorders[9–11]. This similar immune response will be reflected on the dys-regulation of certain critical genes for immune response in blood cells. Due to the high consistency of dys-regulation in different diseases, these genes may serve as reliable biomarkers for a variety of health conditions. Along this line, Gibson and coworkers have proposed a total of 90 “blood informative transcripts” which consist of ten representative genes from each of the nine axes[12]. However, we are interested in finding a much smaller set of genes which can be conveniently assayed with low cost while preserving rich information on health status.

Since much broader range and higher level of gene dys-regulation had been reported in the blood for infectious and autoimmune diseases compared to other diseases, we mainly focused our investigation on infectious and autoimmune diseases in this work. Indeed, we found high consistency of gene dys-regulation for a limited number of genes in infectious and autoimmune diseases. Based on the co-dysregulation pattern in viral and bacterial infections, we selected 20 representative genes, 10 for each category. Common gene dys-regulation in different diseases may suggest similar underlying mechanism of immune response. Furthermore, we demonstrated the potential application of these genes in clinical practice, including disease classification, prognosis and monitoring of treatment effect. In addition, the potential application to the monitoring of personal health may also be of great interest to the general public.

## Materials and methods

### Data collection

We collected public datasets on blood transcriptome from Gene Expression Omnibus (GEO). We mainly focused on microarray datasets on infectious and autoimmune diseases or cancer (solid tumor). For the discovery stage, we collected 20 datasets on infectious or autoimmune diseases for gene selection (**Table A in S1 File**). Only one representative dataset on the whole blood transcriptome was chosen for each disease, preferably with sample size greater than 20 in both case and control groups. These included 4 datasets[13–15] for bacterial infections and 4 datasets[15–18] for viral infections. The remaining 12 datasets[8, 19–29] were related to malaria, systemic lupus erythematosus (SLE), burn, injury, tuberculosis (TB), scleroderma, primary Sjögren’s Syndrome (pSS), rheumatoid arthritis (RA), sarcoidosis, common variable immune deficiency (CVID), Kawasaki disease (KD), systemic-onset juvenile idiopathic arthritis (sJIA). For the validation stage, we collected 34 additional datasets on infectious or autoimmune diseases or cancer (**Table E in S1 File**). For datasets with similar experimental design, we generally selected the ones with larger sample size and higher quality (defined below).

### Quality control

For all the blood transcriptome data, normalization and quality control were performed to filter out the low quality data as follows. The logarithmic values of expression data were first calculated. Next, the probes with greater than 50% missing values or low expression values (< *log*_2_(100)) were removed. Furthermore, for all the remaining data points, the expression data with value below *log*_2_(100) was set to a baseline value *log*_2_(100). Finally, the expression value for genes with multiple probes was calculated as the mean logarithmic value of signal intensity for all probes assigned to the gene.

### Gene selection

For each of the 20 datasets for the discovery stage, we conducted gene differential expression analysis based on rank product after data normalization[30]. We further applied stringent cutoff based on fold change (FC) to select differentially expressed genes (DEGs) with FC>2.0 or FC<0.5. A more lenient cutoff with FC>1.5 or FC<0.7 was also applied to select more genes for consideration. The choice of median over mean in FC calculation was to reduce the effect of genes with large deviation from the group median.

All DEGs in the 20 datasets were ranked by the number of times with FC>2.0 or FC<0.5. We found that down-regulated genes were not as consistently dys-regulated in the 20 datasets as the up-regulated genes. From the top-ranked genes, we further selected 10 genes frequently up-regulated in viral infections, and another set of 10 genes frequently up-regulated in bacterial infections (**Tables B & C in S1 File**). The gene selection procedure is as follows: first, the 55 genes with FC>2.0 or FC<0.5 in at least 8 of the 20 datasets were selected as candidates. For the selection of genes in viral infection, these 55 genes were ranked based on mean FC in the 4 datasets for viral infections and the top 10 genes were selected, all of which turned out to be interferon signaling genes. For the selection of genes in bacterial infections, the 5 genes with mean FC>8.0 in the 4 datasets for bacterial infections and another gene with FC>2.0 in 13 of the 20 datasets were selected. For the genes with mean FC between 5 and 8, only the 5 genes with FC>2.0 in at least 10 of the 20 datasets were considered, which summed up to 11 genes. To deselect one of the bottom two genes with similar mean FC, *TLR5* was chosen over *GYG1* to reflect the importance of TLR signaling pathways in immune response. These 20 genes were then applied to disease classification, prognosis and treatment follow-up.

### Generation the FC matrix of the 20 genes for the 34 validation datasets

In order to classify the diseases in the validation datasets using the 20 genes, we first extracted the expression value of the 20 genes in each dataset. All the samples in these datasets were labeled as either *control* or *case*. The median value of expression within the *control* group was used as the reference for each gene. The choice of median over mean in FC calculation was to reduce the effect of genes with large deviation from the group median. The differential expression value of a gene was defined as the logarithmic expression value subtracted by the reference value of the gene. Next, the FC matrix was generated, the element of which was two to the power of the differential expression value.

### Disease discrimination using K-means models

For the discrimination of viral and bacterial infections, K-means model was used on each dataset (**Table 1**). K-means model which is a popular method for clustering analysis in data mining, aims to partition n samples into k (equals to 2 in our work) clusters. Each sample belongs to the cluster with the nearest mean, serving as a prototype of the cluster. For each dataset in **Table 1**, after the FC matrix was obtained, the number of VRGs with FC>2.0 (NG_V) and the number of BRGs with FC>2.0 (NG_B) were computed. NG_V and NG_B values were combined as a vector to cluster samples by the K-means model. The model was built with ***Scikit-learn[31]***.

**Table 1:** Discrimination of viral and bacterial infections.

*Dataset*	*Factors*	*Model*	*TP*	*FN*	*FP*	*TN*	*Recall*	*Precision*	*F1*
GSE42026	NG_V; NG_B	K-means	33	7	8	11	0.80	0.83	0.81
GSE60244	NG_V; NG_B	K-means	55	1	16	21	0.77	0.98	0.87
GSE72809	NG_V; NG_B	K-means	77	15	18	34	0.81	0.84	0.82
GSE72810	NG_V; NG_B	K-means	27	1	5	18	0.84	0.96	0.90

K-means model was used. TP, true positives; FN, false negatives; FP, false positives; TN, true negatives; F1, the harmonic ratio of *Recall* rate and *Precision* rate. NG_V, number of VRGs with FC>2.0 compared to healthy controls; NG_B, number of BRGs with FC>2.0 compared to healthy controls.

### Disease classification using logistic regression models

Machine learning methods were applied to evaluate the power of the 20 genes in disease classification. Logistic regression was performed on the four diseases with two independent datasets. The standard logistic function is defined as follows:

$$F(x)=\frac{1}{1+e^{-(a_{0}+a_{1}x)}}$$ (1)

Where *a*_0_ and *a*_1_ are decided by training data. For instance, in the burn disease, the FC matrixes of datasets GSE37069 and GSE19743 were obtained and the x in the above model was the FC value of *HP*. The model was fit by the FC values of *HP* of all samples in training data (GSE37069) to decide *a*_0_ and *a*_1_. Then, we classified the test data GSE19743 by the fitted model. This model was built with ***Scikit-learn[31]***, a third party library for Python.

### Measures for classification and clustering

Several measures were used to assess the power of a classification or discrimination model described above, including precision, recall and F1 as defined below,

Precision: $p=\frac{TP}{TP+FP}$
Recall: $R=\frac{TP}{TP+FN}$
F1: $\frac{2}{F1}=\frac{1}{P}+\frac{1}{R}$ or $F1=\frac{2*TP}{2*TP+FP+FN}$

Where TP was *true positive*, FP was *false positive*, TN means *true negative* and FN means *false negative*. In our work, F1 was adopted to balance the contribution from precision and recall.

### Pearson correlation coefficient for co-expression and age effect

To study the co-expression relationship among the top genes, we selected 55 genes with FC>2.0 in at least 8 of the 20 discovery datasets (**Table B in S1 File**). We calculated the Pearson correlation coefficients (PCC) among these 55 genes in dataset GSE48348. The co-expression network with PCC>0.50 among these 55 genes was drawn by software ***Cytoscape***. Pearson correlation coefficient was also adopted when we calculated the relationship between gene expression and age. The build-in correlation function in **R** language was used.

### The effect of sex on gene dys-regulation

To study genes dys-regulation in different sexes, fold changes in different sexes were calculated separately. After quality control, we obtained the fold changes of the 20 genes from the expression matrix for the whole datasets without considering the gender information. We also separated the samples into male and female groups and calculated the FC within those two groups.

### Personalized health monitoring

Aligned sequencing reads in bam format were downloaded from the GEO database (GSE32874). Then raw reads counts were calculated using ***HTSeq*** python package. Variance stabilizing transformation implemented in **R** package ***DESeq2*** was performed on raw reads counts to produce the final gene expression value. The expression values of the 20 genes were extracted and plotted in **R**.

## Results

### Genes frequently dys-regulated in infectious and autoimmune diseases

First, we collected public datasets on blood transcriptome for infectious and autoimmune diseases. Based on the quality of the datasets, we selected one representative dataset for each disease (whole blood only, **Table A in S1 File**). For these 20 datasets, we first conducted differential expression analysis (patients compared to healthy controls). Then, we applied a stringent cutoff for fold change (FC>2.0 or FC<0.5) to reduce the number of significant genes. The significant genes were ranked based on the frequency of dys-regulation in these 20 datasets (**Table B in S1 File**). We found that certain genes displayed high frequency of significant dys-regulation in these diseases with the vast majority being up-regulation.

**Fig 1:**
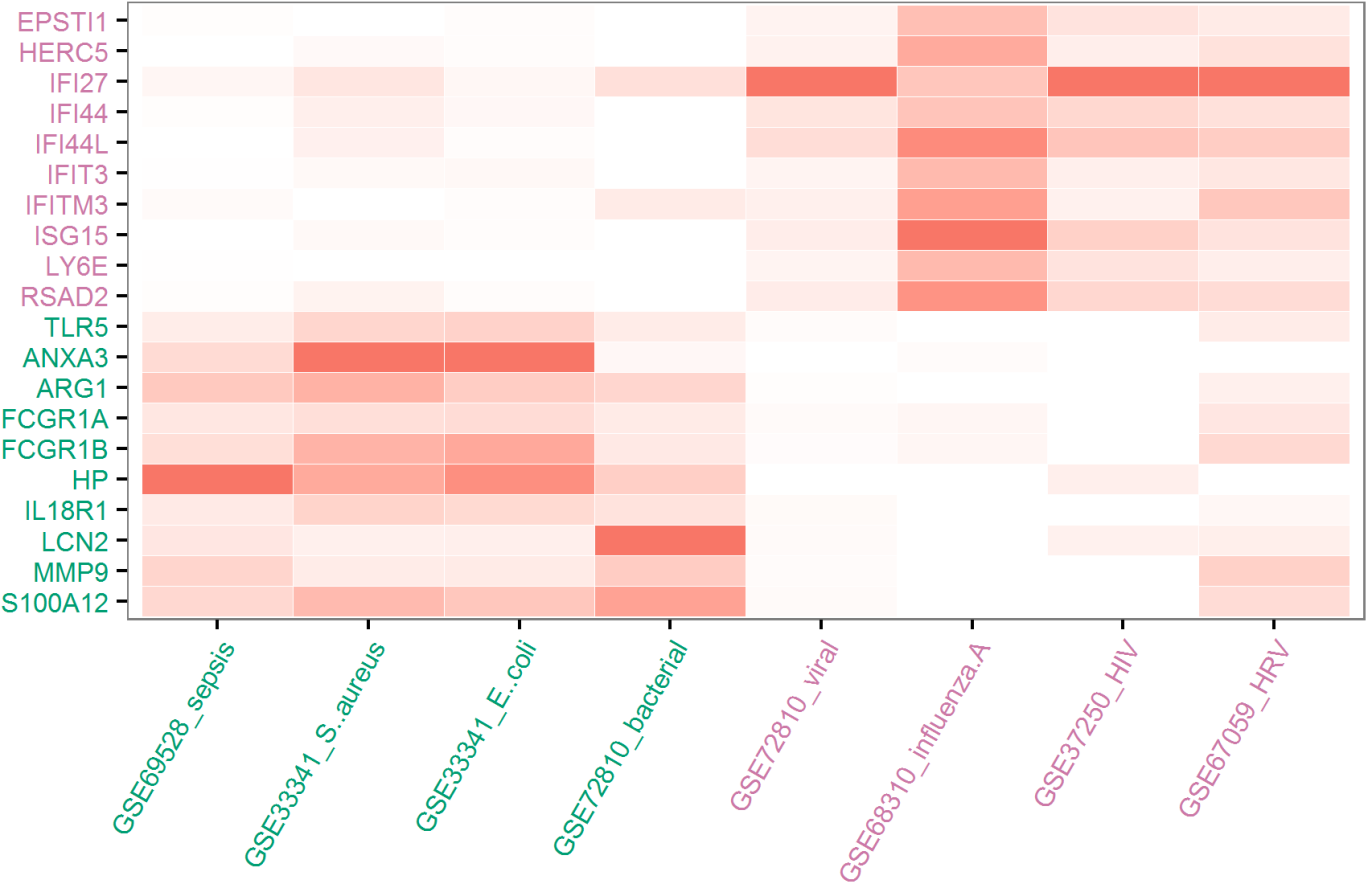
The most significantly dys-regulated genes in viral and bacterial infections. Darker color indicates larger fold change. For more details regarding the datasets, please refer to **Table A in S1 File**. For more details on the fold change values, please refer to **Table C in S1 File.**

For further gene selection, we targeted a total number of 20 genes that could be conveniently assayed with low cost. Since both viral and bacterial infections displayed high within-group concordance, we decided to select genes based on the co-dysregulation pattern in viral and bacterial infections. This ensured that the selected genes were functional connected rather than being unrelated biomarkers. The 20 datasets included 4 clearly defined viral infections and 4 bacterial infections. Considering the mean fold change in viral or bacterial infections, the total number of significant dys-regulation among the 20 datasets, and direction of gene dys-regulation, we selected 10 genes consistently up-regulated in viral infections and another set of 10 genes up-regulated in bacterial infections (**Fig 1, Table C in S1 File,** please refer to the **Methods** section for the detailed procedure for gene selection). The rankings were relatively lower for the consistently down-regulated genes. In addition, these genes did not display clear distinction between viral and bacterial infections. Therefore, the down-regulated genes were not considered for further analysis.

The genes up-regulated in viral infections were all involved in interferon signaling, including *IFI27*, *IFI44L* and *ISG15*. On the other hand, the genes up-regulated in bacterial infections such as *HP*, *ANXA3* and *ARG1* were annotated as anti-bacterial response but were involved in diverse pathways. For the ten virus-response genes (VRGs), the average fold change was between 4.97 and 17.42 in viral infections. For the ten bacteria-response genes (BRGs), the average fold change was between 4.90 and 15.91 in bacterial infections. Collectively, both VRGs and BRGs were specific to the corresponding infection type because these genes had much lower FC in the other type of infection.

**Fig 2:**
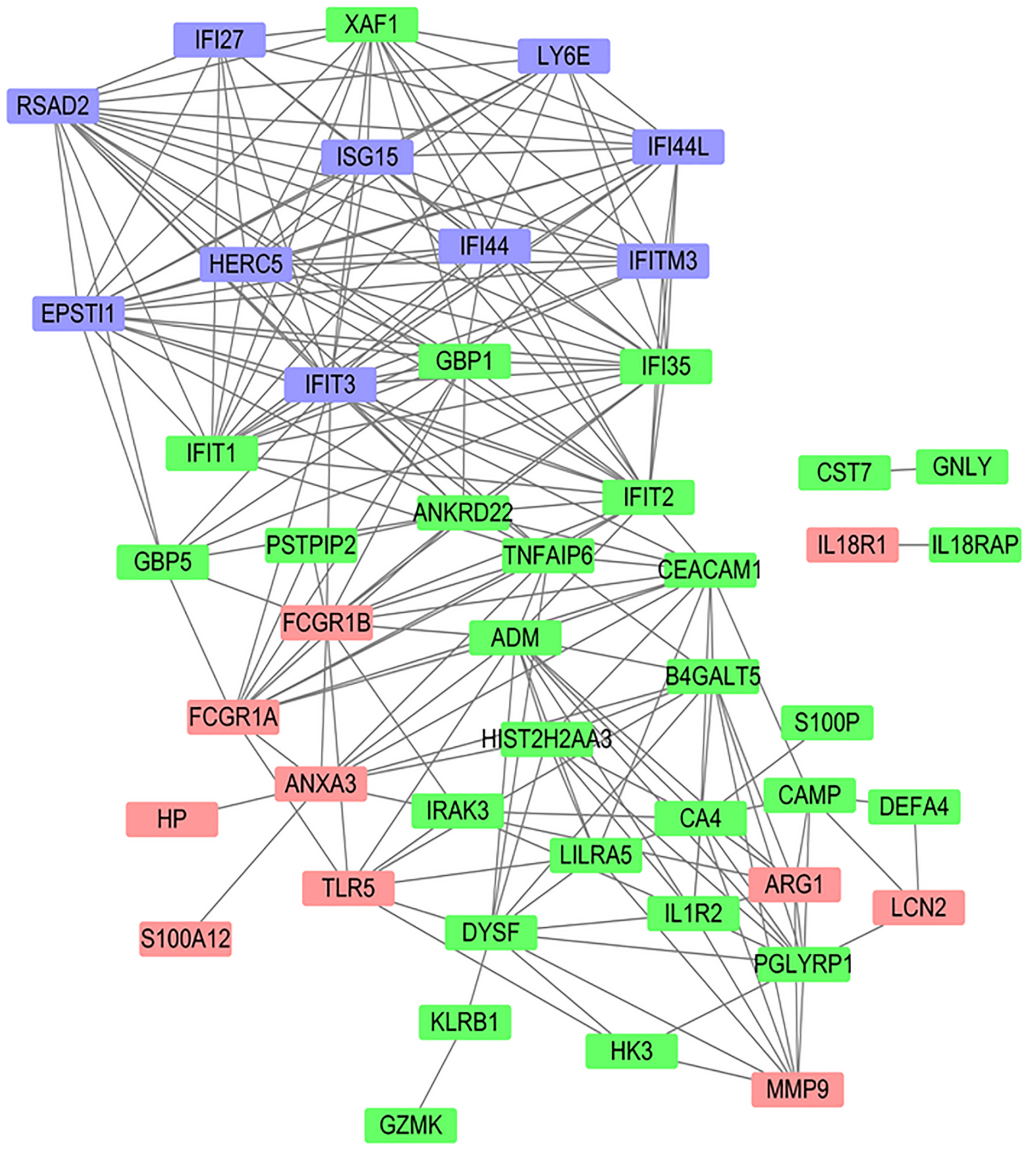
A co-expression network for the top genes in infectious and autoimmune diseases. The 55 genes with fold change (FC>2.0) in at least 8 of the 20 discovery datasets were selected for co-expression analysis. The dataset GSE48348 with 734 blood samples was used to construct the co-expression network. A cutoff 0.50 for Pearson correlation coefficient was used to retain only strong connections among these 55 genes. Blue color indicates the 10 selected virus response genes (VRGs), while pink color indicates the 10 selected bacteria response genes (BRGs).

To further demonstrate the functional connections among the 20 genes, we conducted co-expression analysis on the top 55 genes with FC>2.0 in at least 8 of the 20 datasets (**Table B in S1 File**). We found that most of these 55 genes were strongly connected to each other. The co-expression network could be roughly divided into two sub-networks (**Fig 2**), one containing all of the 10 VRGs and the other containing 9 of the 10 BRGs. The BRGs may be further divided into two sub-groups, one containing *ARG1*, *MMP9* and *LCN2* and the other containing *HP*, *S100A12*, *ANXA3*, *TLR5*, *FCGR1A* and *FCGR1B*.

For the other 12 diseases among the 20 discovery datasets, we also observed clear up-regulation of either VRGs or BRGs (**Table D in S1 File**). Tuberculosis (TB) was the only disease with significant up-regulation of both VRGs and BRGs, albeit showing lower consistency in BRGs including much higher level of up-regulation of *FCGR1A* and *FCGR1B* compared to other BRGs. In sarcoidosis and common variable immune deficiency (CVID), moderate up-regulation of VRGs was observed, accompanied by the up-regulation of *FCGR1A* and *FCGR1B* in the BRG category. Up-regulation of BRGs was observed in Kawasaki disease (KD) and systemic juvenile idiopathic arthritis (sJIA) with notable exception of *FCGR1A* and *FCGR1B*. Considering all 20 discovery datasets, *FCGR1A* and *FCGR1B* seemed to be robust makers for respiratory tract infections, including both viral and bacterial infections.

Among these 12 diseases, SLE displayed the most significant up-regulation of VRGs, accompanied by moderate up-regulation of a few BRGs. In both burn and injury, significant up-regulation of BRGs was observed, which could be partially induced by secondary infection after burn or injury. In addition, down-regulation of several VRGs were observed in burn and injury, which was clearly different from other diseases examined here. Significant up-regulation of VRGs was also observed in scleroderma and primary Sjögren's Syndrome (pSS). On the other hand, significant up-regulation of BRGs was observed in malaria. In addition, moderate up-regulation of a few BRGs was also observed in rheumatoid arthritis (RA). Overall, these 20 VRGs and BRGs were frequently dys-regulated in many infectious and autoimmune diseases. The distinctive patterns observed here may deserve further investigation in future studies.

We shall note that we did not observe much gene dys-regulation in some of the datasets we examined for infectious or autoimmune diseases, including uremia, psoriasis, ankylosing spondylitis, and chronic obstructive pulmonary disease (COPD). Due to the limited number of datasets available for these diseases, we may need to wait for more datasets with high quality before a convincing conclusion can be drawn for these diseases.

### Potential applications of these 20 genes as biomarkers for infectious and autoimmune diseases

#### Viral vs bacterial infections

To test whether these 20 genes could be used in distinguishing viral infections from bacterial infections, we collected four datasets containing both types of infections. As a simple test, we used the number of significant gene dys-regulation in VGRs and BGRs to discriminate viral from bacterial infections (**Table 1**). It was clear that high accuracy of classification could be achieved in these four datasets with F1 values ranging from 0.81 to 0.90 (the definition of F1 value can be found in **Methods**). Further exploration with these 20 genes may lead to discrimination models with higher accuracy.

For comparison, we also summarized the relevant findings from the original publications of the datasets. In two of the original studies, a 156-transcript signature and a 10-gene signature were identified for the discrimination of viral and bacterial infections, respectively[32] [33]. In another original study on children, a 2-gene signature was derived which included *IFI44L* and *FAM89A*[15]. *FAM89A* was not among our top genes from the 20 discovery datasets.

#### SLE vs bacterial infections

Due to the high level and consistent up-regulation of VRGs in SLE, it’s relatively easy to separate SLE from bacterial infections and autoimmune diseases with the gene dys-regulation pattern of bacterial infections (**Table 2**). The F1 value ranged from 0.86 to 0.93 in the three datasets where SLE was compared against JIA or staphylococcus. As comparison, discrimination between SLE and other diseases was not the focus in the three original studies[22, 25, 34].

**Table 2:** Discrimination of SLE and other diseases.

*Dataset*	*Factors*	*Model*	*TP*	*FN*	*FP*	*TN*	*Recall*	*Precision*	*F1*
GSE17755	NG_V; NG_B	K-means	50	7	1	21	0.98	0.88	0.93
GSE29536	NG_V; NG_B	K-means	86	10	18	49	0.83	0.90	0.86
GSE22098	NG_V; NG_B	K-means	78	4	7	45	0.92	0.95	0.93

K-means model was used. Please refer to Table 1 for the meanings of the abbreviations. SLE has the gene dys-regulation pattern of viral infections. Thus, NG_V and NG_B can be used to distinguish SLE from bacterial infections or certain autoimmune diseases with the gene dys-regulation pattern of bacterial infections. GSE17755: SLE vs JIA. GSE29536: SLE vs sJIA. GSE22098: pediatric SLE vs pediatric staphylococcus.

#### Single gene as biomarker

To test whether single-gene could be used as biomarker for infectious and autoimmune diseases, we collected four diseases each with two independent datasets. We found that the discrimination model derived from one dataset can be applied to another dataset with high accuracy (**Table 3**). For sepsis, burn and injury, the expression level of *HP* could separate patients from healthy controls with F1 value of 0.99–1.00. For Kawasaki disease, the expression level of *ANXA3* could separate patients from healthy controls with F1 value of 0.97.

**Table 3:** Single gene as biomarker for infectious or autoimmune diseases.

*Disease*	*Training Data*	*Test Data*	*Gene*	*TP*	*FN*	*FP*	*TN*	*Recall*	*Precision*	*F1*
*Burn*	GSE37069	GSE19743	HP	112	2	1	62	0.99	0.98	0.99
Sepsis	GSE69528	GSE80496	HP	24	0	0	21	1.00	1.00	1.00
*Injury*	GSE36809	GSE11375	HP	155	3	0	26	1.00	0.98	0.99
KD	GSE63881	GSE68004	ANXA3	75	1	4	33	0.95	0.99	0.97

Logistic model was used. KD, Kawasaki disease. Please refer to Table 1 for the meanings of the abbreviations.

As comparison, simple biomarker was not the focus of the original publications for these datasets. [15], [20], [35], [36], [21], [28].

#### Biomarker for HIV-1 infection

**Fig 3:**
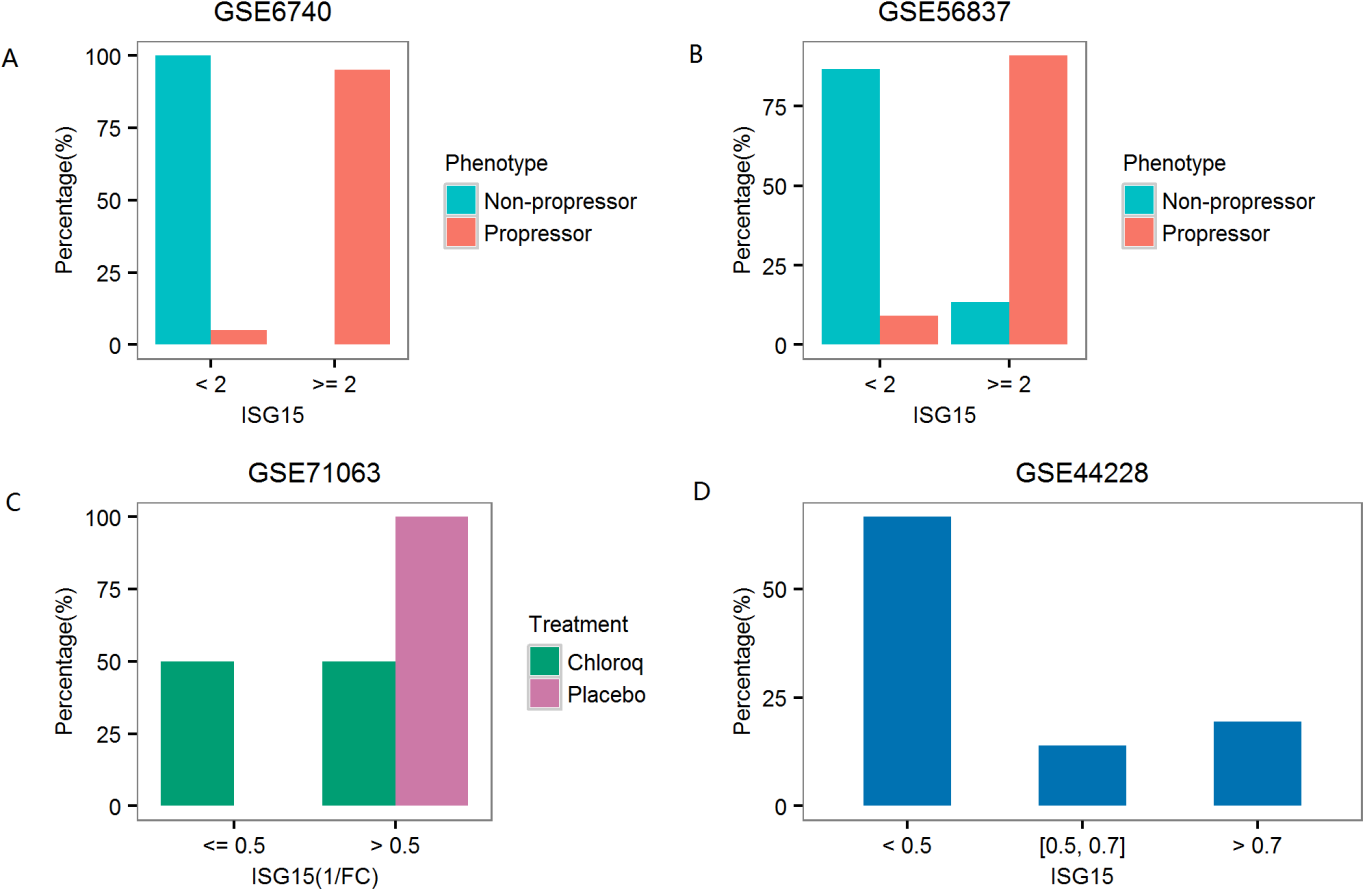
Single-gene biomarker for HIV-1 infection. (A), (B) Progressors and non-progressors of HIV-1 infection can be distinguished using the expression of *ISG15*. (C,), (D) Treatment effect can also be monitored using the expression of *ISG15*. In dataset GSE44228, Samples were after treatment with antiretroviral therapy (ART). In the upper panel, fold induction was calculated against the median level of healthy controls. In the lower panel, fold reduction was calculated against the pre-treatment level of the same patient.

Infection of HIV-1 virus leads to significant up-regulation of VRGs (**Table C in S1 File**). We found that *ISG15* could serve as a reliable biomarker for HIV-1 infection in several independent datasets (**Fig 3**). Using the expression value of *ISG15*, progressors and non-progressors could be classified with high accuracy. In the two datasets examined, 91–95% of the progressors had *ISG15* level above 2-fold of median value among the controls, while it’s below the threshold for 87–100% of the non-progressors. The expression level of *ISG15* could also be used in monitoring the effect of drug treatment. Drug treatment led to 2-fold reduction of *ISG15* level in 50–67% of the patients, while the use of placebo did not lead to 2-fold reduction of *ISG15* level in any patients.

As comparison, simple biomarker was not the focus of the original publications of these datasets[37]. [38]. [39].

#### Biomarker for TB

**Fig 4:**
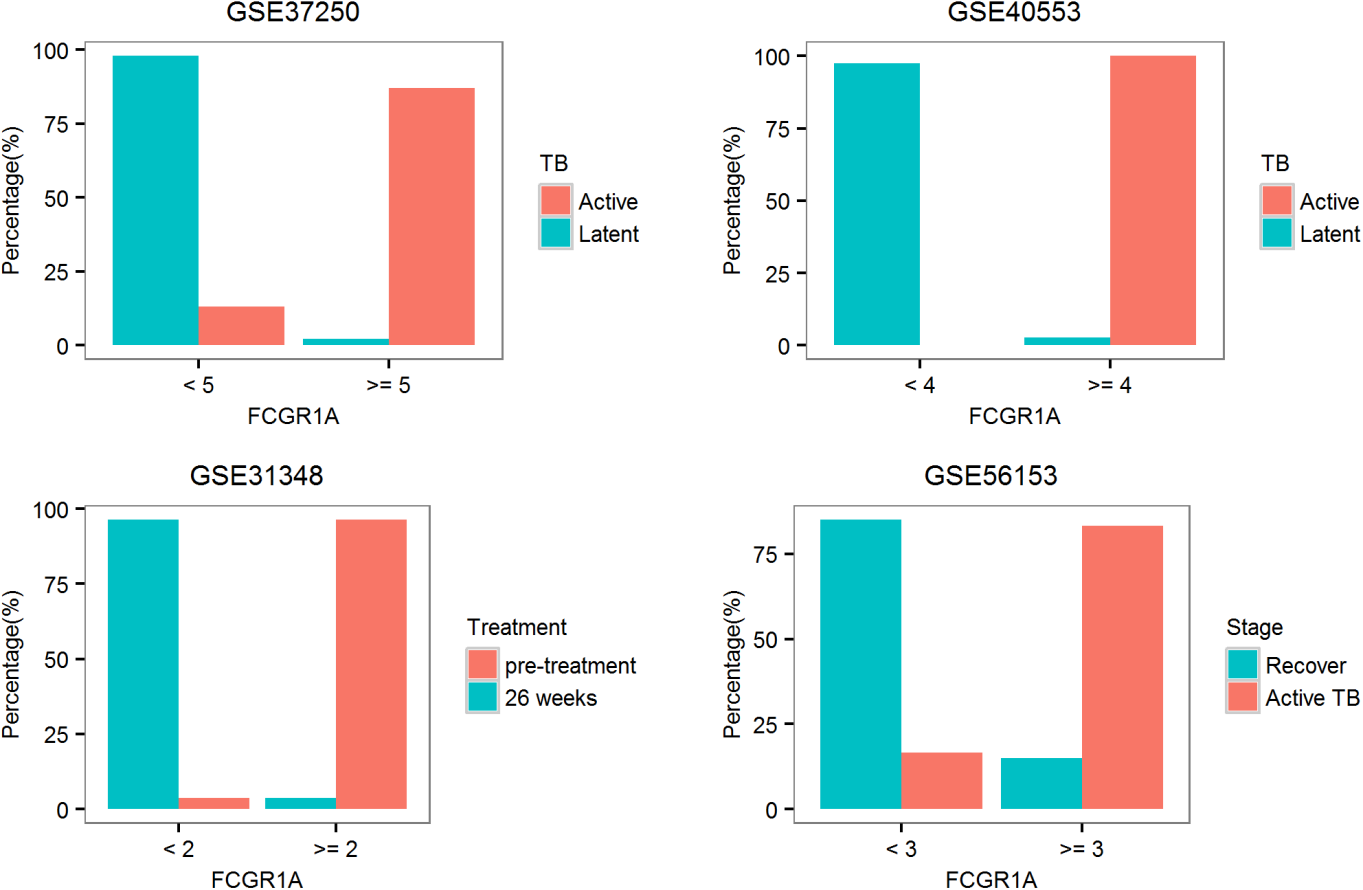
Single-gene biomarker for TB. Active and latent TB can be distinguished using the expression of *FCGR1A*. Treatment effect can also be monitored using the expression of *FCGR1A*. Fold induction of *FCGR1A* was calculated against the median level of healthy controls.

As stated earlier, TB is a unique disease with significant dys-regulation of both VRGs and BRGs. We found that *FCGR1A* from BRGs could serve as a reliable biomarker for TB in several independent datasets (**Fig 4**). Using the expression value of *FCGR1A*, active and latent TB could be classified with high accuracy. In the two datasets examined, 87–100% of the patients with active TB had high level *FCGR1A* expression, while only 2–3% of the patients with latent TB had high level *FCGR1A* expression using the same cutoff. The expression of *FCGR1A* could also be used in monitoring the effect of drug treatment. Significant reduction of *FCGR1A* level in 85–96% of the patients was observed after a long-period of drug treatment. We shall note that the different cutoff applied here is not uncommon in microarray studies which are originated from different laboratories using different assay platforms and different experimental designs.

As comparison, one of the original studies proposed a 27-gene signature including *FCGR1A* and *FCGR1B* for the discrimination of active and latent TB[18]. In another original study, a 664-transcript signature was proposed for the discrimination of active and latent TB [40]. In the original studies on the treatment response, large number of signature genes were normalized during the treatment [41]. [42].

#### Biomarker for sJIA

**Fig 5:**
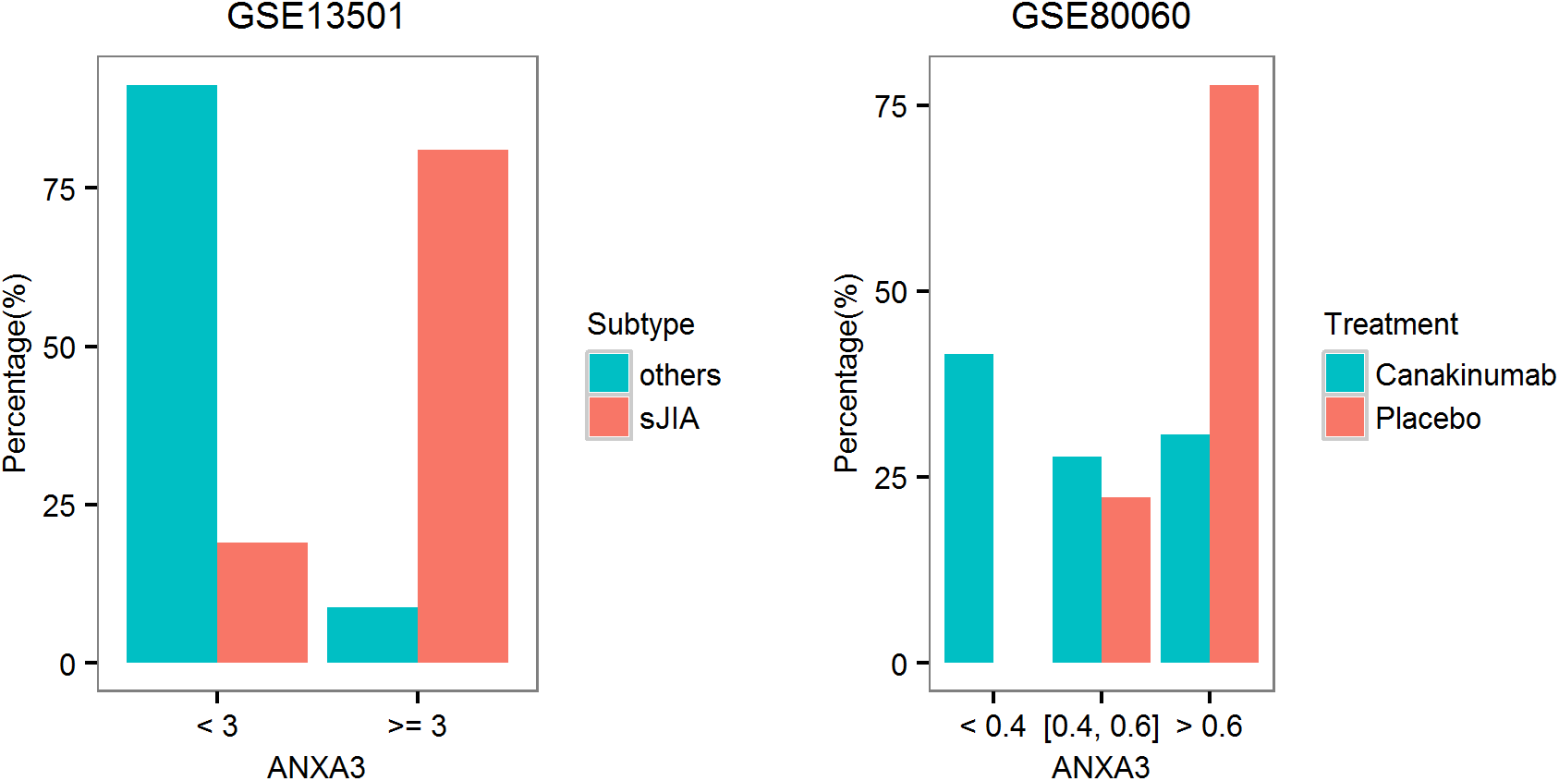
Single-gene biomarker for JIA. Systemic and non-systemic JIA can be distinguished using the expression of *ANXA3*. Treatment effect can also be monitored using the expression of *ANXA3*. In the left panel, fold induction was calculated against the median level of healthy controls. In the right panel, fold reduction was calculated against the pre-treatment level of the same patient.

Systemic JIA is accompanied by the up-regulation of BRGs. We found that *ANXA3* could serve as a reliable biomarker for sJIA in independent datasets (**Fig 5**). Using the expression level of *ANXA3*, systemic and non-systemic JIA could be classified with high accuracy. In the dataset examined, 81% of the sJIA patients had *ANXA3* level above 3-fold of the median value among the controls, while it’s below the threshold for 91% of non-systemic JIA patients. The expression of *ANXA3* could also be used in monitoring the effect of drug treatment. Significant reduction of *ANXA3* level (2.5 fold) in 42% of the patients was observed after drug treatment, while it’s not observed in any of the patients treated with placebo.

As comparison, the original studies did not focus on simple biomarker for sJIA [43].

### Potential biomarkers for stroke and cancer

#### Biomarker for stroke

**Fig 6:**
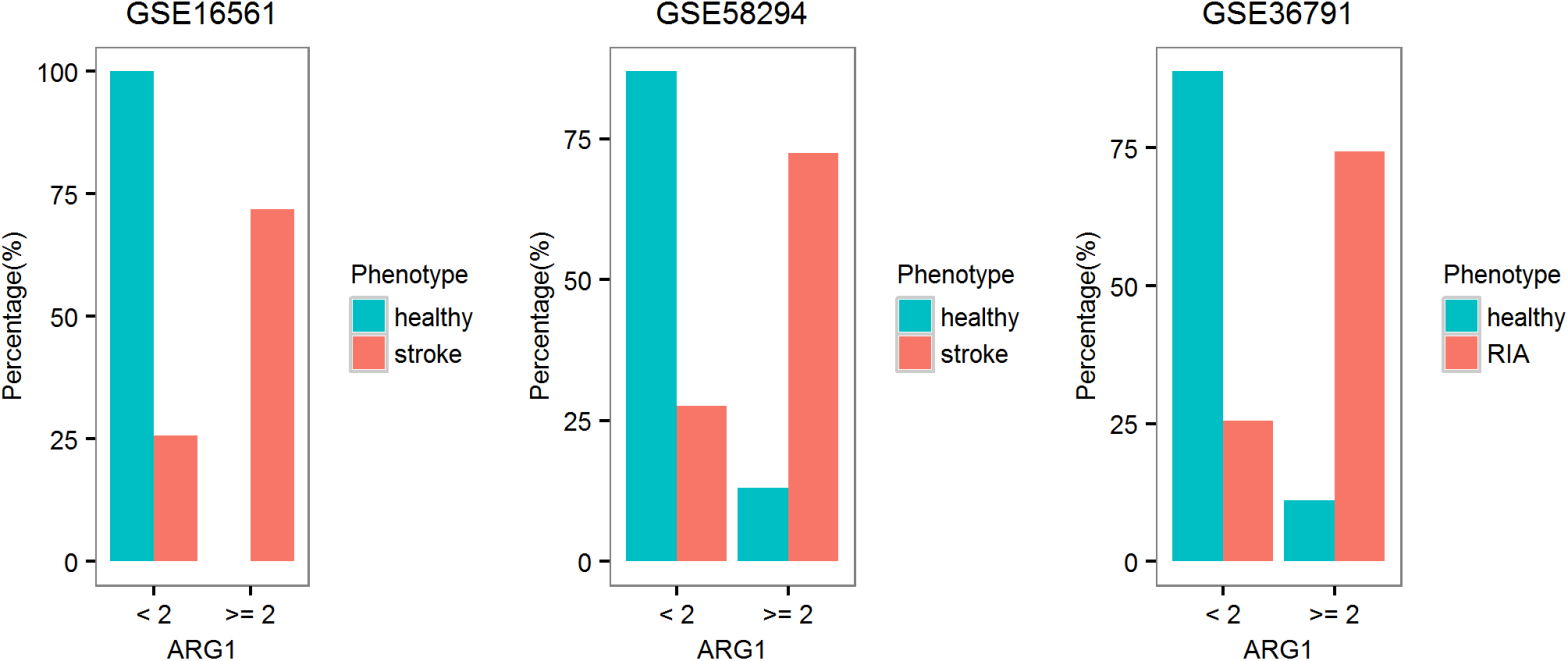
Single-gene biomarker for stroke and RIA. Most of the patients with stroke or RIA displayed 2-fold induction of *ARG1* expression. RIA, ruptured intracranial aneurysms.

The potential of single gene as biomarker for diseases was not limited to infectious and autoimmune diseases. We found consistent dysregulation of BRGs in stroke and ruptured intracranial aneurysms (RIA). Among the BRGs, the expression level of *ARG1* could separate patients from controls with high accuracy (**Fig 6**). More details for the selection of *ARG1* could be found in **Table F in S1 File** (the selection procedure for single-gene biomarker was similar for other diseases in this work). In these three datasets, 72–74% of the patients had *ARG1* level above 2-fold of the median expression value among the controls, while it was below the threshold for 87–100% of the controls, resulting in F1 value of 0.82–0.84. In addition, *MMP9* displayed similar level of accuracy as single-gene biomarker. Thus, *ARG1* and *MMP9* may be used in monitoring the recovery from stroke.

As comparison, in one of the original studies on stroke, a 9-gene signature was derived, including *S100A12*, *ARG1* and *MMP9* of our BRGs[44]. Other original studies did not focus on biomarker[45].

#### Biomarker for cancer

**Fig 7:**
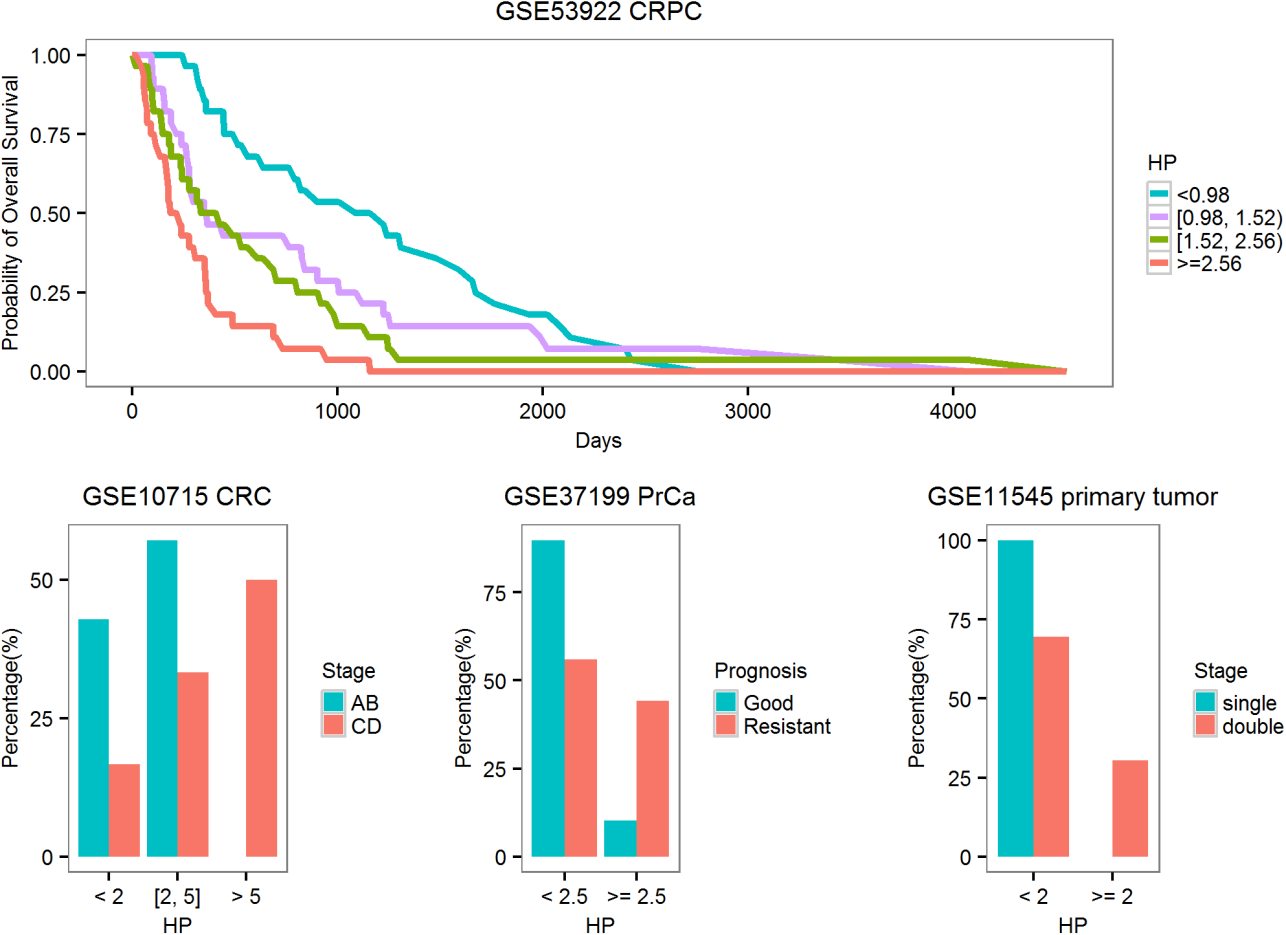
Single-gene biomarker for cancer. The expression of *HP* could be used in the disease classification and prognosis in several independent datasets for cancer. The expression level of *HP* was indicated as its induction level using the median expression level in healthy controls as the reference. CRPC, castration-resistant prostate cancer. CRC, colorectal cancer. PrCa, prostate cancer.

Cancer can also lead to up-regulation of immune response in peripheral blood. We found that *HP* may serve as a biomarker for cancer progression based on several independent datasets (**Fig 7**). For example, a 5-fold induction of *HP* indicated the advanced stage in colorectal cancer (50% of the patients at stage CD while none at stage AB). A 2-fold induction of *HP* indicated double primary tumor in another dataset. The expression of *HP* could also be used in prognosis. In two independent datasets for prostate cancer, higher expression of *HP* indicated poor prognosis. Distinctive survival curves were observed for patients with different levels of *HP* expression.

As comparison, the original study on colorectal cancer mainly focused on the discovery of biomarkers from the tissue sample[3]. In the original study of double primary tumors, the authors focused on the discovery of 9 probes with statistically significant expression between single and double primary tumors, none of which was among the 20 genes described in this work[46]. In one of the original studies on prostate cancer, a nine-gene signature was proposed for prognosis, none of which is among the 20 genes described in this work[4]. In the other original study on prostate cancer, the expression levels of 35 genes including *HP* and *LCN2* were found to be correlated with overall survival (OS). In addition, the rs5472 of HP was also found to be correlated with OS[47].

### Application to personal health monitoring

**Fig 8:**
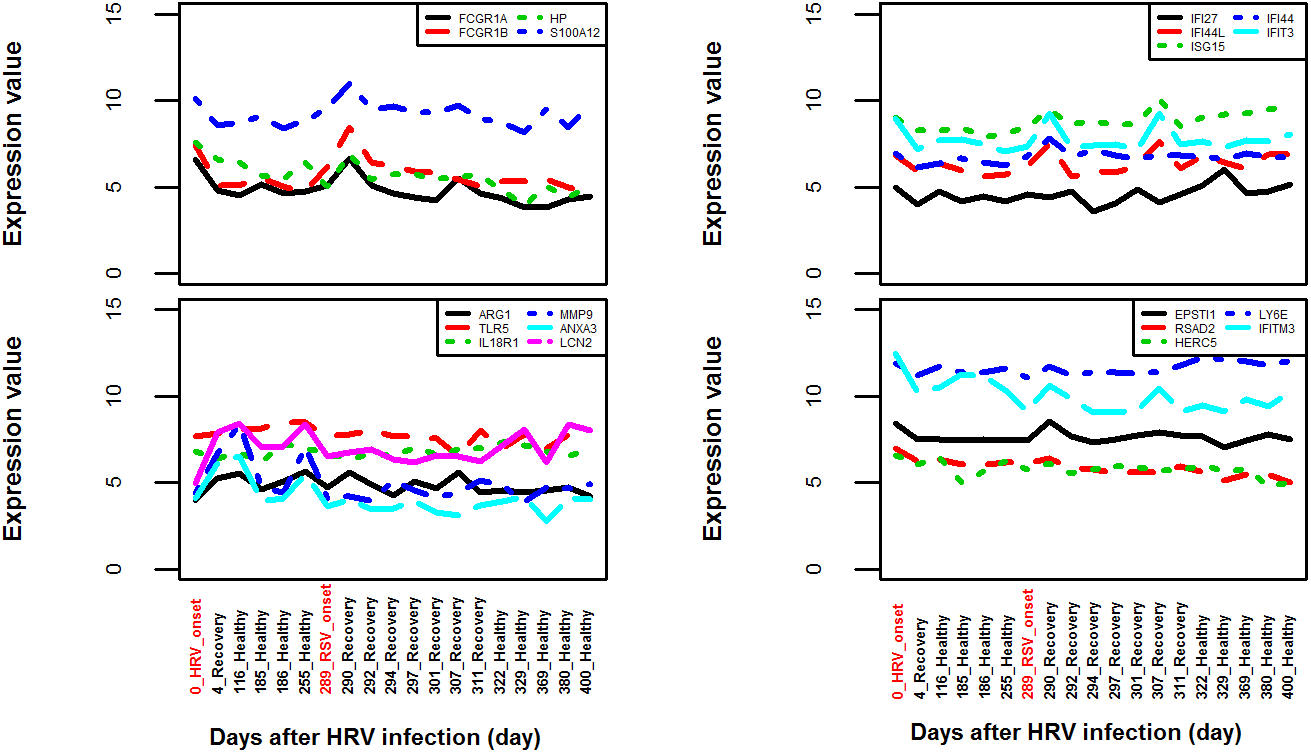
Use of the 20 selected genes in personal health monitoring. The expression values of these 20 genes were extracted from an RNA-seq dataset GSE32874 which contains the multi-point sampling of the blood transcriptome of a single man over a period of 400 days. The HRV onset and RSV onset events were both captured by the dys-regulation of multiple VGRs as well as several BRGs. The expression profiles also captured an unknown event at day 301 discussed in the original publication.

Continuous health monitoring is critical for the early detection of health risks. In a pioneering study on personalized medicine, multiple omics technologies were applied to the continuous data collection from a single man over a period of 14 months[48]. Two viral infection events were recorded during the period, one HRV infection and another RSV infection. We extracted the expression value of the 20 VRGs and BRGs and plotted the longitudinal expression profiles. The two infection events could be clearly detected from the expression levels of multiple VRGs and BRGs (**Fig 8**). The expression levels were clearly higher during infection and came down at the recovery periods. Interestingly, an “unknown event” at day 301 discussed in the original publication was also clearly detectable on the expression profiles of multiple VRGs and BRGs. As comparison, it was not detected by the CRP level and only came to light after sophisticated analysis of the blood transcriptome data. The local peak at day 307 coincided with the sudden elevation of blood glucose and onset of diabetes. Due to the much lower cost of assaying 20 genes than whole transcriptome profiling, this set of 20 genes may have potential to be applied to personal health monitoring.

### Evaluating the robustness and confounding factors

**Fig 9:**
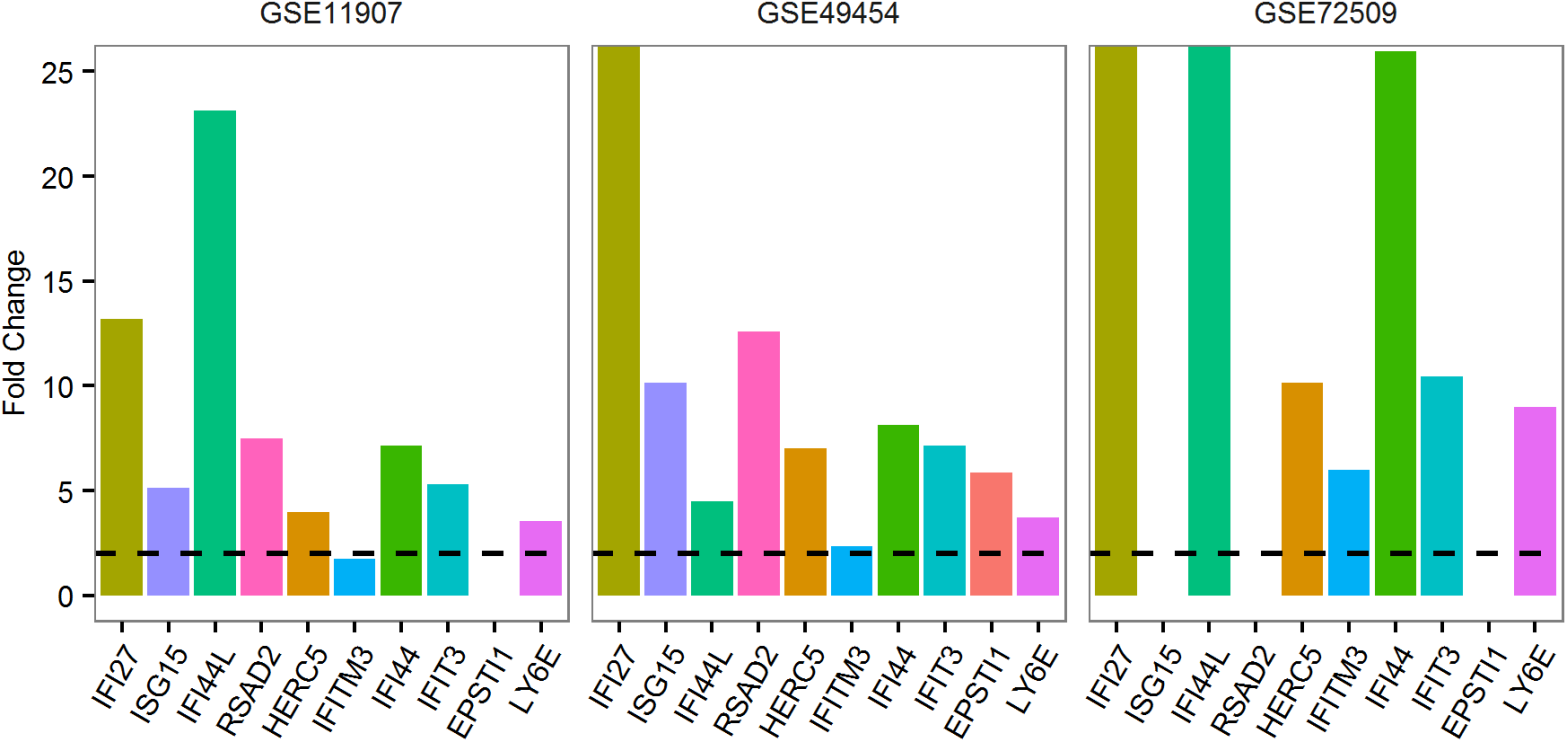
Consistent gene dys-regulation pattern in SLE. Significant up-regulation of VRGs was observed in whole blood (middle) and PBMC (left). Consistent pattern was also observed in microarray (middle and left) and RNA-Seq (right) platforms.

SLE is one of the few diseases with many high quality datasets. Thus, it’s a good candidate disease to test the robustness of the 20 genes. In the whole blood of SLE, we observed significant up-regulation of VRGs, some of which were above 10 fold (**Fig 9**, middle set). This feature was replicated in the peripheral blood mononuclear cell (PBMC, left set). In addition, the pattern observed in microarray studies was also replicated in an RNA-Seq study (right set). Therefore, the gene dys-regulation pattern described in previous sections may not be limited to whole blood or microarray platform.

Studies on tissues with multiple cell types may be complicated by the change of cell composition[49, 50]. It is likely that some of the above-described dysregulation may be partially attributed to the change of blood cell composition. Nevertheless, similar feature have been observed in several diseases with different blood cell components. For example, both whole blood and PBMC in SLE displayed similar up-regulation of VRGs (**Fig 9**). In HIV-1 infection, we also observed distinctive expression pattern of VRGs in progressors and non-progressors in independent datasets on whole blood or CD4+ and CD8+ T cells (**Fig 3**). Although more detailed information can be obtained by conducting assays on sorted blood cells, it may be sufficient to simply examine the whole blood for many diseases.

To study the effect of gender on gene dys-regulation in the blood, we examined two datasets with gender information (**Table J in S1 File**). It’s well known that SLE is heavily biased to female. In the dataset GSE65391, both male and female SLE patients displayed significant dys-regulation of the 20 genes especially the VRGs. However, the level of dys-regulation in female patients was much higher than that in male patients (**Table K in S1 File**). Nine of the 20 genes exceeded 1.4 fold on the ratio of gene dys-regulation. On the other hand, Kawasaki disease (KD) is slightly biases to male. In the dataset GSE63881, both male and female KD patients displayed significant dys-regulation of the 10 BRGs. However, no significant difference could be found on the gene dys-regulation between male and female patients (**Table K in S1 File**). Therefore, the effect of sex on gene dys-regulation seems to be different for different diseases. Both sexes could be analyzed separately when the gender information is available and sample size is sufficiently large.

To study the effect of age on blood transcriptome, we examined four additional datasets with age information (**Table J in S1 File**). Overall, the correlation of individual gene expression with age was weak. Only *NELL2* displayed negative correlation with age in all four datasets (from -0.29 to -0.47) (**Table L in S1 File**). In addition, five genes displayed negative correlation with age in three of the four datasets, including *CCR2*, *CCR7*, *MYC*, *LTB* and *FAM102A*. The genes with positive correlation with age were not as consistent in the four datasets. Overall, the 20 selected genes described in this work did not show even marginal correlation with age in any of the four datasets examined here.

## Discussion

### Functional relevance of the 20 genes to human health

Genetic variations in some of these 20 genes have been reported to be associated with the susceptibility to various diseases. Among the BRGs, *ANXA3* is associated with rheumatoid arthritis in Japanese[51]. *HP* is associated with several diseases and longevity[52]. *FCGR1B* is associated with breast cancer[53]. *MMP9* is associated with metaphyseal anadisplasia 2. Among the VRGs, *ISG15* is associated with immunodeficiency 38. *IFI44L* is associated with psychiatric disorders including schizophrenia and bipolar disorder[54]. *IFI44L* is also associated with febrile seizure caused by measles, mumps and rubella vaccination[55]. It’s interesting that some of these genes are also the most reliable single-gene biomarkers for the diseases analyzed in this work.

In addition to the dys-regulation of mRNAs described in this study, the dys-regulation of proteins in serum, plasma and primary tissues has also been reported in the literature. For examples, haptoglobin level in the plasma was a good prognostic biomarker for acute myocardial infarction[56]. In ovarian cancer, the expression of annexin A3 corresponded to the resistance to platinum treatment[57]. In sickle cell disease, arginase activity in the plasma was found to be significantly increased[58]. In chronic heart failure, plasma level of S100A12 was significantly elevated[59]. Plasma level of MMP-9 was also an independent risk factor for first time coronary heart disease[60]. In combination with the dys-regulation of gene expression described in this work, the functional relevance of these 20 genes to human health has been supported by three levels of evidence, including genetic variations, dysregulation of mRNAs, and dysregulation of proteins.

According to the modular framework proposed by Pascual V and coworkers[61], 8 of the 10 VRGs belong to module 3.1 which is enriched with interferon-inducible genes (**Table 4**). In fact, the other two genes *IFI27* and *ISG15* are also well-established interferon-inducible genes. Five of the 10 BRGs belong to module 2.2 which is enriched with neutrophil marker genes. In addition, *S100A12* is assigned to module 3.3 with broad definition of inflammation.

**Table 4:** Module assignment and relevant functions of the 20 genes.

*Gene*	*Module ID*	*Relevant Function*
EPSTI1	3.1	IFN signaling
HERC5	3.1	IFN signaling
IFI27		IFN signaling
IFI44	3.1	IFN signaling
IFI44L	3.1	IFN signaling
IFIT3	3.1	IFN signaling
IFITM3	3.1	IFN signaling
ISG15		IFN signaling
LY6E	3.1	IFN signaling/immune regulator
RSAD2	3.1	IFN dependent and independent response
TLR5		Bind to flagellin/activate NFKb pathway
ANXA3	2.2	Calcium and phospholipid
ARG1	2.2	ARG metabolism/immune response
FCGR1A		phagocytosis
FCGR1B		phagocytosis
HP	2.2	Antioxidant activity / binding to free Hb
IL18R1		IL signaling
LCN2	2.2	Stabilize MMP9/bind to ferric siderophore
MMP9	2.2	Matrix degradation
S100A12	3.3	Bing to RAGE/activate NFKb/inhibit MMP9

According to our data analysis presented in this work, host response to viral infections seems to converge to the activation of interferon signaling pathway upon detection of viral RNA in the cytoplasm. On the other hand, host response to bacterial infections involves much more complex mechanism. *FCGR1A* and *FCGR1B* can recognize microbial wall components and induce phagocytosis. *ANXA3* has also been identified in phagosome upon bacterial infection, likely involving its calcium and phospholipid biding ability. *LCN2* can bind to ferric siderophore and restrict the critical nutrient for bacteria. *TLR5* can bind to flagellin and activate NFKb pathway. Matrix degradation protein *MMP9* seems to play a special role in host response because both *LCN2* and *S100A12* can interact with *MMP9*. *IL18R1* is a critical component of the interleukin signaling pathways. In addition, *ARG1* can metabolize arginine which is an immune regulator.

Viral and bacterial infections are among the biggest enemies of human health. During the long history of evolution, humans have developed defense system to fight against invasions from virus and bacteria. This defense system may also be utilized to resolve problems arising from within the human body. Some of the autoimmune diseases are likely caused by unknown infections, therefore it’s not surprising to observe similar immune response. It’s also understandable that similar immune response is observed in cancer, stroke and some other non-infectious diseases.

### Disease-specific genes in peripheral blood

We mainly explored commonly dys-regulated genes in the blood of various disease. It is also of vital importance to find out genes only dys-regulated in a specific disease. We used SLE as an example to examine this issue due to the availability of multiple high quality datasets (**Table G in S1 File**). We found that the 10 VRGs were still among the top-ranked genes in the nine datasets for SLE (**Table H in S1 File**). Some other top-ranked genes in SLE were also dys-regulated in multiple datasets of other diseases examined in this study. Therefore, we did not observe a single gene specifically dys-regulated in SLE with high confidence. In fact, we found that it was difficult to identify disease-specific genes for viral infections and autoimmune diseases with the gene dys-regulation pattern of viral infections in the original 20 discovery datasets.

We then used sepsis as another example to examine the issue of disease-specific gene. Among the three datasets for sepsis (**Table G in S1 File**), we found 141 genes consistently dys-regulated in all three datasets (**Table I in S1 File**). Based on the low frequency of dys-regulation in the 20 discovery datasets, some of the genes such as *TMCO3* could potentially serve as disease-specific markers for sepsis. With the increasing availability of high quality datasets for various diseases, this issue of disease-specific genes may be re-examined in the future. In a recent work, deconvolution of cell composition was applied to the blood transcriptome data for the discovery of disease-specific genes[62]. Again, multiple high quality datasets will be needed for the validation of the proposed disease-specific markers.

### Utilities of house-keeping genes in peripheral blood

House-keeping genes have been frequently used as internal reference in experiments such as RT-qPCR. We found that many genes were stably expressed in the disease datasets examined in this study, including the well-known house-keeping genes *ACTB*, *B2M*, *UBC* and *GUSB*. We explored whether the relative expression level of the 20 genes as compared to the house-keeping genes can be used in disease classification, prognosis and treatment evaluation. As examples, we used *B2M* as the internal control in the datasets of TB and cancer. We found that the relative expression level of the biomarker gene achieved similar performance without using the healthy controls as reference. This is important because the selection of healthy controls could be problematic as observed in some datasets examined in this study.

### Individual response to stress and treatment

We observed that individuals may respond differently to certain stress, as evidenced by the large variation of the induction of the 20 genes in many diseases. Some of the variation could be linked to the expression level at the baseline. We also observed heterogeneity in individual response to treatments. For example, patients with HIV-1 infection responded differently to anti-viral treatment (**Fig 3**). The expression of *ISG15* was reduced over 2-fold in some patients but not others. We found that patients with higher level of *ISG15* expression generally had better response in terms of the reduction of *ISG15* expression. This could also be explained in another way. For those patients with relatively low level of *ISG15* expression, it may not be necessary to further reduce its expression level. Thus, the expression profile of these 20 genes prior to stress and treatment shall be considered when evaluating the individual response to stress and treatment.

### Limitations of the current study

The main limitations of this study include the limited high quality datasets for each disease and limited sample size for each dataset. In an ideal scenario, there are multiple high quality datasets for each disease and every dataset has at least 100 samples for each study group (such as case and control groups). However, this requirement can not be fulfilled at the current stage, which may affect the ranking of high frequency gene dys-regulation. To partially alleviate the problem, we restrict our selection of datasets to only those of direct human sources. This means that the blood samples were profiled without any *in vitro* treatments. Blood transcriptome studies on animal models were also excluded entirely. Nonetheless, some hidden factors may still exert effect on the data analysis. For examples, the use of antibiotics, smoking, alcohol drinking, and insufficient sleep may all confound the data analysis. The selection of only commonly dys-regulated genes under diverse health conditions may partially reduce the noise but certainly not all. In sum, we share bear these limitations in mind when evaluating the results presented in this work.

## Conclusion

In this work, we have demonstrated that a small set of 20 genes displayed frequent and significant dys-regulation in infectious and autoimmune diseases. Biomarkers based on these commonly dys-regulated genes are likely more robust and reproducible than those biomarkers from a few limited studies. In combination with other disease-specific assays, these genes may assist in disease classification, patient stratification, prognosis and treatment follow-up. These genes may also be used in personal health monitoring. More works are warranted to explore its application in clinical practice and to elucidate the exact role of each gene in immune response. In practice, we would suggest the selection of a few VRGs and BRGs to monitor immune dys-regulation, in addition to the selection of a few potential disease-specific genes from previous studies. This way we could investigate at medium to large scale how the immune system is involved in the disease mechanism and whether certain VRGs or BRGs could become biomarkers for the disease.

## Supporting information

S1 File Major expression datasets and lists of selected differentially expressed genes, Table **A**, The discovery datasets: 20 datasets for infectious or autoimmune diseases. Table **B**, List of all dys-regulated genes in the 20 discovery datasets. Table **C**, Dys-regulation of the 20 genes in the 8 datasets of viral or bacterial infections. Table **D**, Dys-regulation of the 20 genes in the 12 datasets for infectious or autoimmune diseases. Table **E**, The validation datasets: additional 34 datasets on infectious and autoimmune diseases or cancer. Table **F**, Performance of single-gene biomarkers with the 20 genes on the 3 datasets of cerebrovascular diseases. Table **G**, Datasets for investigating disease-specific genes in SLE and sepsis. Table **H**, Top-ranked genes in SLE. Table **I**, Top-ranked genes in sepsis. Table **J**, Datasets for the evaluation of gender and age effect. Table **K**, Dys-regulation of the 20 genes in SLE and KD for different genders. Table **L**, Genes with weak correlation with age in 4 datasets.
(XLSX)
